# A new genus and species of Laelapidae from Iran with notes on *Gymnolaelaps* Berlese and *Laelaspisella* Marais & Loots (Acari, Mesostigmata)

**DOI:** 10.3897/zookeys.549.6891

**Published:** 2016-01-05

**Authors:** Alireza Nemati, Dariusz J. Gwiazdowicz

**Affiliations:** 1 Department of Plant Protection, Faculty of Agriculture, University of Shahrekord, Iran; 2 Poznan University of Life Sciences, Faculty of Forestry, Wojska Polskiego 71C, 60–625 Poznań, Poland

**Keywords:** Taxonomy, *Pogonolaelaps* gen. n., ant nest, *Gymnolaelaps*, *Laelaspisella*

## Abstract

*Pogonolaelaps* **gen. n.** a new genus of Laelapidae Berlese is erected and described based on *Pogonolaelaps canestrinii* (Berlese), **comb. n.** as well as on specimens which were collected from soil and ant nests in different parts of Iran. Also, a new species *Pogonolaelaps beaulieui* **sp. n.** is described based on specimens collected from soil, litter and ant nests in various parts of Chaharmahal va Bakhtiari province, Iran. The genus *Gymnolaelaps* is revised considering species with some morphological deviations. *Gymnolaelaps reniculus* (Karg, 1981) and *Gymnolaelaps triquetrus* (Karg, 2003) are removed from this genus and placed in their original genus *Pseudoparasitus*. The characters of *Laelaspisella* Marais & Loots, 1969 are discussed in a detail along with a proposal of a new definition.

## Introduction

The Laelapidae Berlese comprises a multitude of morphologically and behaviorally diverse mite groups that are free-living or associated with arthropods, mammals and birds (e.g. Strong and Halliday 1994, Faraji and Halliday 2009, Lindquist et al. 2009). Hypotheses concerning the evolutionary history of this family and its relatives are minimally developed and the classification of the group is consequently inadequate (Casanueva 1993). This family is considered to include different subfamilies, genera and subgenera by different authors (Berlese 1903, 1923, Baker and Wharton 1952, Van Aswegen and Loots 1970, Karg 1978, 1979, 1982, 1993, 2000, Krantz and Ainscough 1990, Casanueva 1993, Krantz 1998). Lindquist et al. (2009) considered nine subfamilies for laelapid mites, based in part on a phylogenetic study of free-living and arthropod-associated taxa by Casanueva (1993), and the recently characterized subfamily Acanthochelinae (Radovsky and Gettinger 1999).

*Gymnolaelaps* was considered at different taxonomic levels: as a subgenus of *Hypoaspis* sensu lato (Berlese 1916, Evans and Till 1966, Hunter 1967), as a subgenus of *Pseudoparasitus* (Karg 1989, 1993) and as a distinct genus (Sellnick 1935, Evans and Till 1979, Joharchi et al. 2011; Joharchi and Halliday 2013). We herein consider *Gymnolaelaps* as a genus. The genus *Gymnolaelaps* has been collected in many parts of the world, mainly from ants’ nests. The genus includes approximately 35 described species (Joharchi et al. 2011). So far, a total of eight species notwithstanding their validity were reported from Iran: *Gymnolaelaps canestrinii* (Berlese), *Gymnolaelaps kabitae* Bhattacharyya, *Gymnolaelaps laevis* (Michael), *Gymnolaelaps messor* Joharchi et al., *Gymnolaelaps myrmecophilus* (Berlese), *Gymnolaelaps myrmophilus* (Michael) and *Gymnolaelaps prestoni* Joharchi et al., *Gymnolaelaps artavilensis* Joharchi & Halliday (Joharchi and Halliday 2013).

*Laelaspisella* was erected by Marais and Loots (1969) in Laelapinae to accommodate two new species: *Laelaspisella macrodorsalis* Marais & Loots and *Laelaspisella epigynialis* Marais & Loots collected from forest soil in South Africa, Lesotho and Congo. Subsequently, this genus was considered as a subgenus of *Hypoaspis* sens. lat. by Karg (1989), who described two additional species, Hypoaspis (Laelaspisella) foramenis Karg, 1989 and Hypoaspis (Laelaspisella) cavitatis Karg, 1982 and as distinct genus by Joharchi and Halliday (2013). The latter authors considered *Laelaspisella canestrinii* and *Laelaspisella kabitae* as two more species of this genus but excluded two mentioned species of Karg of *Laelaspisella* (Joharchi and Halliday 2013).

During our survey on Mesostigmata mites inhabiting soil and litter, *Pogonolaelaps canestrinii* (Berlese), comb. n. from different habitats and localities was discovered. Based on that species, a new genus, *Pogonolaelaps* gen. n. is proposed and described. Also, a new species of *Pogonolaelaps* found in Iran is described. Redescription of *Pogonolaelaps canestrinii* comb. n. is given along with additional information on the specimens of this species in Berlese collection. The definition of *Laelaspisella* is also revised.

## Materials and methods

Mites were collected from soil and litter samples from different parts of Iran. Mites were extracted using Berlese funnels, placed in lactic acid at 55 °C for clearing and then mounted in Hoyer’s medium on permanent microslides. Line drawings were made by the use of a drawing tube and figures were elaborated with Corel X-draw software, based on the scanned line drawings. All measurements are given in micrometers (μm). The dorsal setae notation, leg and palp chaetotaxy follows that of Lindquist and Evans (1965), Evans (1963a, b) and Evans and Till 1965 respectively. Terminology for idiosomal glands and lyrifissures follows Johnston and Moraza (1991). We have attempted to identify all pore-like structures, but we acknowledge that some may have been overlooked. The holotype and some of the paratypes (four females and two males) of the new species are deposited in the Acarological Laboratory, Department of Plant Protection, Agricultural College, Shahrekord University (APAS), Shahrekord, Iran. Three female paratypes are deposited in the Senckenberg Museum fur Naturkunde Görlitz, Am Museum, Görlitz, Germany; Natural History Museum Cromwell Road London SW7 5BD UK; and National Museum of Natural History (NMNH), Smithsonian Institute Washington D.C., 20013-7012 USA. One female paratype and one male are deposited in Poznan University of Life Sciences, Department of Forest Pathology Wojska Polskiego 71C, 60–625 Poznań, Poland. Redescription of *Pogonolaelaps canestrinii* comb. n. was based on the specimens in Acarological Laboratory, Department of Plant Protection, Agricultural College, Shahrekord University Shahrekord, Iran, which the figures have been compared with type specimens in the Berlese collection (Italy) by Dr. Roberto Nanelli.

## Systematics

### Genus Pogonolaelaps gen. n.

Taxon classificationAnimaliaMesostigmataLaelapidae

http://zoobank.org/94CC5068-5F2C-4222-8D3B-54DD32D3216D

#### Type species.

*Laelaps canestrinii* Berlese, 1903, by original designation.

#### Genus diagnosis.

Female with a three-tined palp tarsal claw; the dorsal seta of the chelicerae present, epistome smooth, corniculi horn-like, internal malae free medially and densely fringed with very elongate hairs, in addition possess two detachments of densely and very elongate hairs at basal part of each internal mala; *st*4 absent; genital shield large, abutting anal shield, with *st*5 on shield and three pairs of setae adjacent to the lateral edges; scimitar-shaped dorsal setae with small knob at their base, dorsal shield with holotrichous status on podonotal and hypertrichous on opisthonotal part, plus 0-3 unpaired setae between *J* series and 7-9 pairs of long tick setae on latero-posterior part of opisthonotal region; genu and tibia I with normal chaetotaxy (2 3/2 3/1 2), and genu IV with ten setae including two ventral setae (*av* and *pv*). Male with separate anal shield and strong spine-like seta on femur II.

#### Description.

***Idiosomal dorsum*.** Dorsal shield oval shaped, well sclerotized, nearly wraps around and overlaps onto the ventral idiosoma, podonotal part with holotrichous and opisthonotal with hypertrichous condition, shield with 51-55 pairs of setae, 28-32 pairs on opisthonotal region, plus 0-3 *Jx* setae between *J* series (usually with 3), *rx* seta present on podonotal part, setae increasing in length from anterior to posterior and from dorso-central to dorso-lateral part, latero-posterior part of opisthonotal region with 7-9 pairs of long, thick, barbed setae (Figs 11, 15), dorsal setae with a small basal knob (Fig. 17). Dorsal shield generally with six pairs of large slit-like lyrifissures (Figs 11, 15).

**Figures 1–6.**
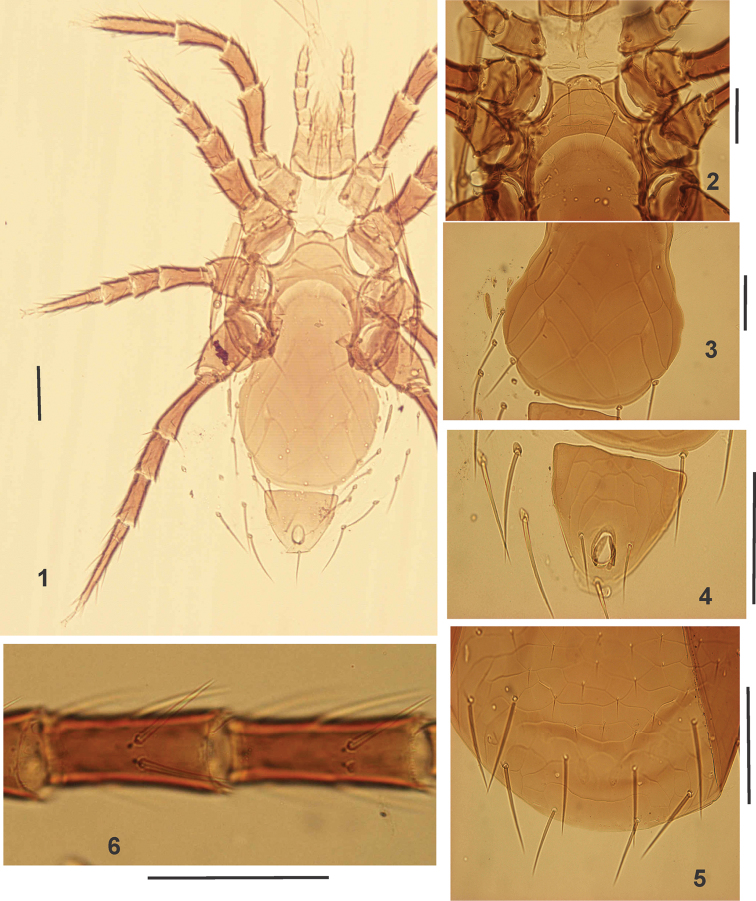
*Pogonolaelaps canestrinii* (Berlese). (Female): **1–2** Ventral idiosoma **3** Epigynal shield **4** Anal shield **5** End part of dorsal shield **6** Genu and tibia IV (ventral view). Scale bars: 100 µm.

***Idiosomal venter*.** Tritosternum with columnar base and paired free pilose laciniae; pre-sternal plates weakly sclerotized and ornamented with transverse lines (Figs 1, 2, 12, 16a). Sternal shield widest between coxae II and III, anterior margin sinuate, convex medially, posterior margin deeply concave (Figs 1, 2, 16a). With three slit-like *iv1-3*, all located on the surface of sternal shield (Fig. 16a); *st4* absent (Figs 2, 16a). Endopodal plates II/III fused to lateral margins of sternal shield, endopodal plates III/IV elongate, curved. Large crescent-shape podal plates surrounding coxae IV, fused with contiguous exopodal plates and extended to the anterior level of coxae II (Figs 1, 16a).

**Figures 7–10.**
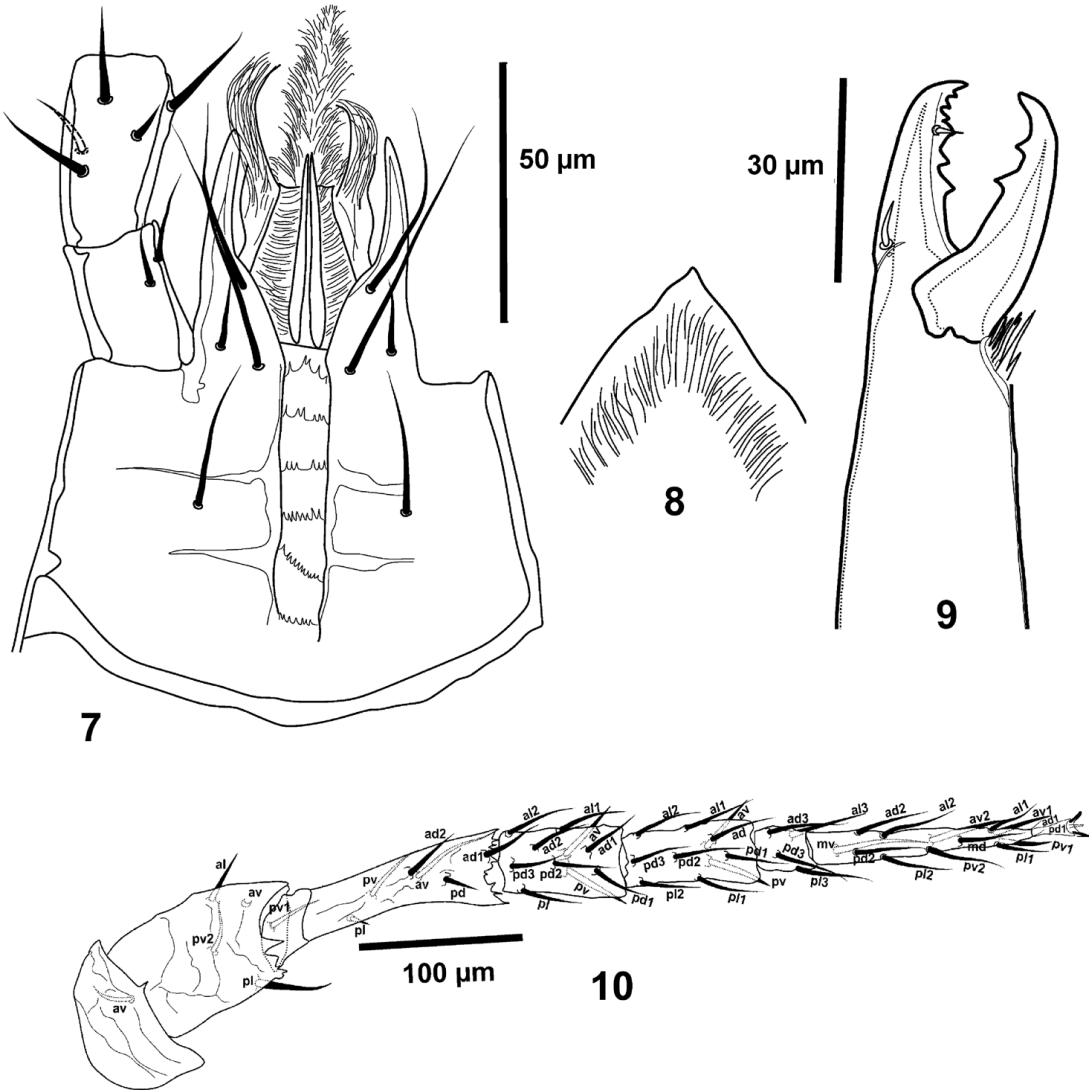
*Pogonolaelaps canestrinii* (Berlese). (Female): **7** Hypostome **8** Epistome **9** Chelicera **10** Leg IV.

**Figures 11–14.**
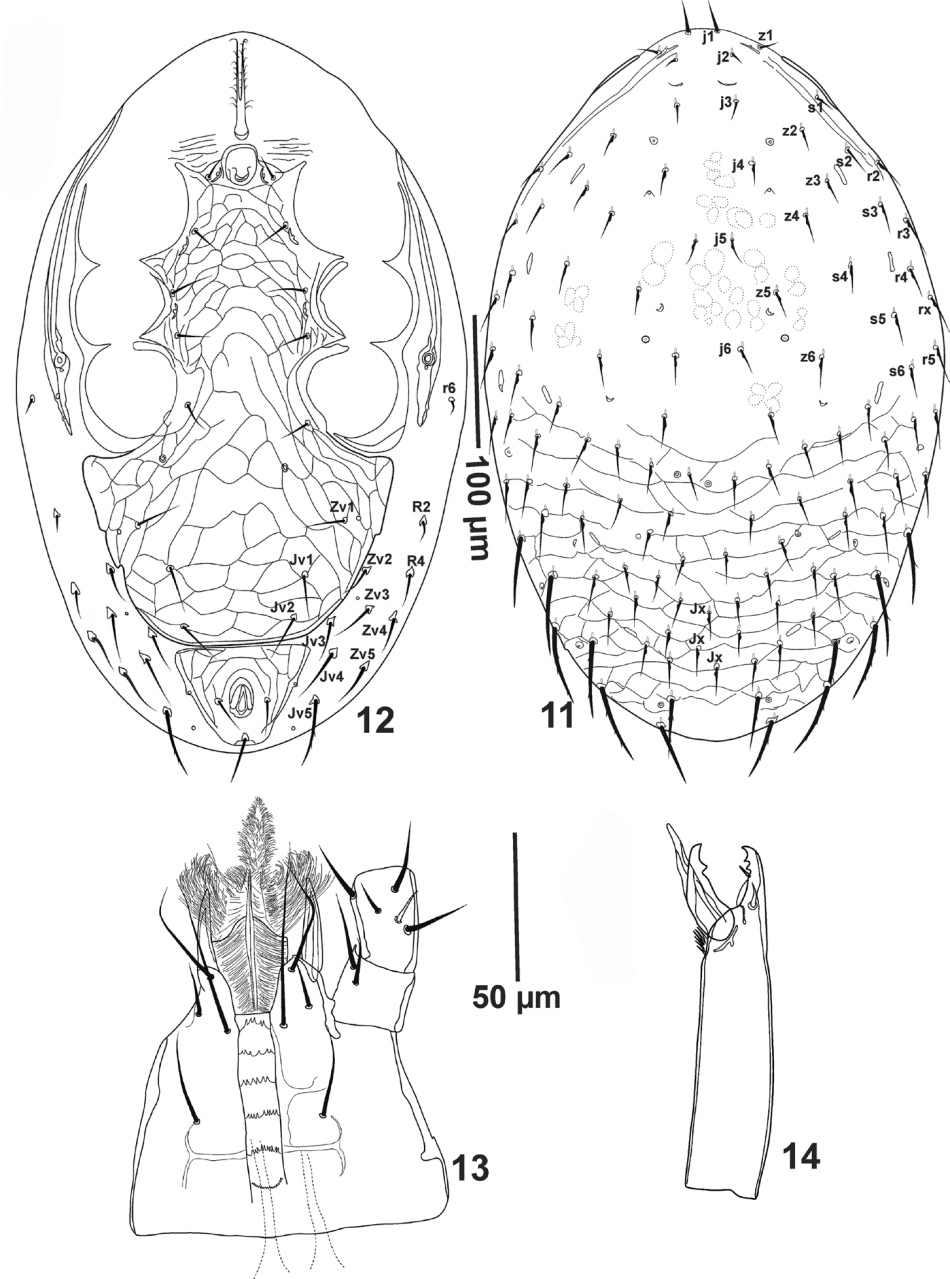
*Pogonolaelaps canestrinii* (Berlese). (Male): **11** Dorsal shield **12** Ventral idiosoma **13** Hypostome **14** Chelicera.

**Figures 15–17.**
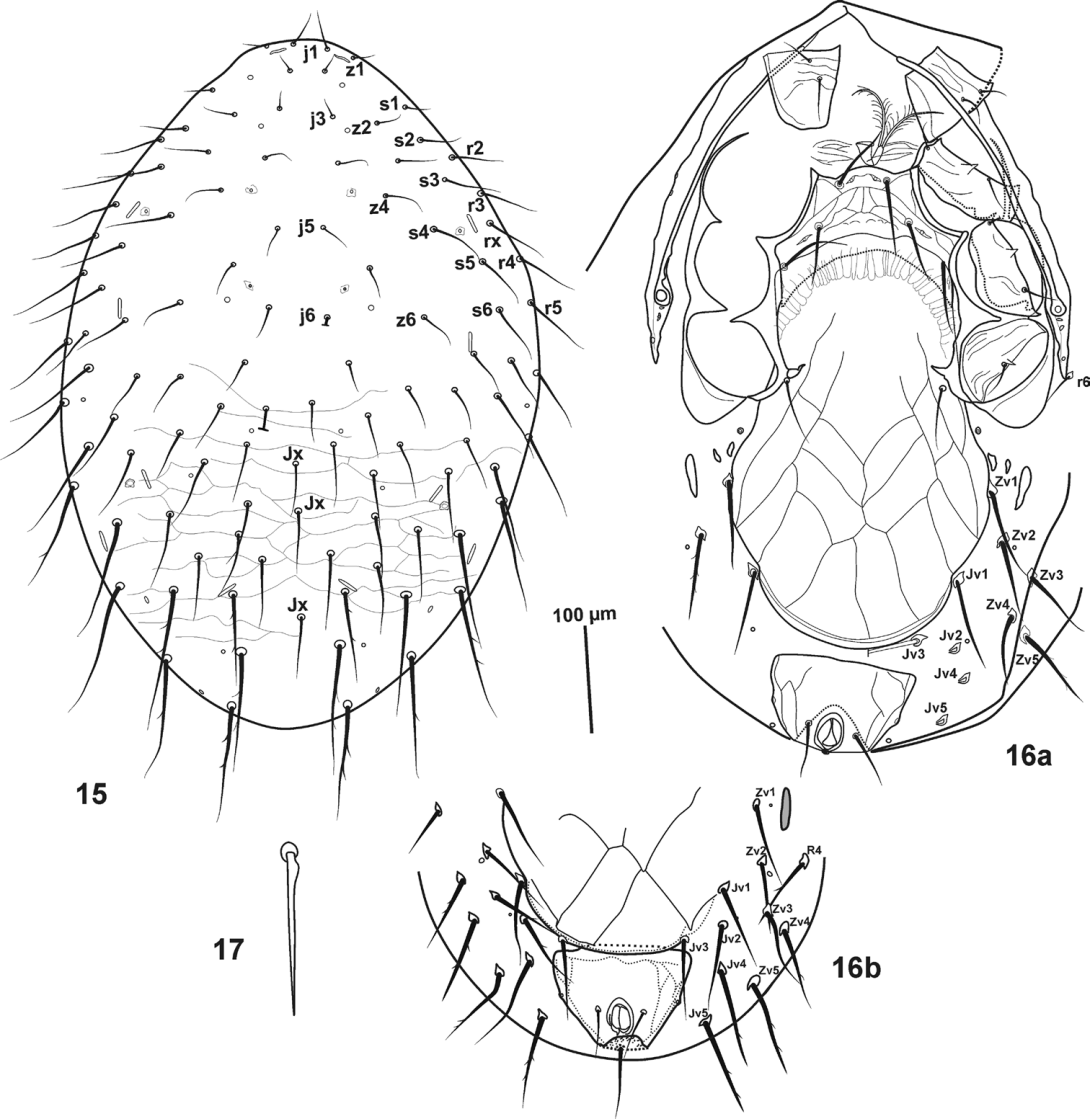
*Pogonolaelaps beaulieui* Nemati & Gwiazdowicz, gen. n., sp. n. (Female): **15** Dorsal shield **16a, b** Ventral idiosoma **17** An example of dorsal shield setae.

Genital shield broad, abutting anal shield, with one pair of setae (*st5*) on shield and three pairs of setae adjacent to lateral edges (Figs 1, 3, 16a); circular paragenital pores (*iv5*) located on soft integument between coxa IV and pair of minute narrow platelets (Figs 1, 16a). Anal shield subtriangular. Opisthogastric surface with: one pair of elongate metapodal plates, two pairs of minute platelets, 10-11 pairs of long setae, *r6*, *Jv1-5* and *Zv1-5* usually present. Peritreme extending from coxa IV to anterior level of coxa I (Fig. 16a), peritrematal shield wide, with two pairs of post-stigmatal pores, one pair of small pores anterior to stigmata and two pairs of pores (*ip*, *gp*) at level of coxae II/III (Fig. 16a at left side).

**Figures 18–22.**
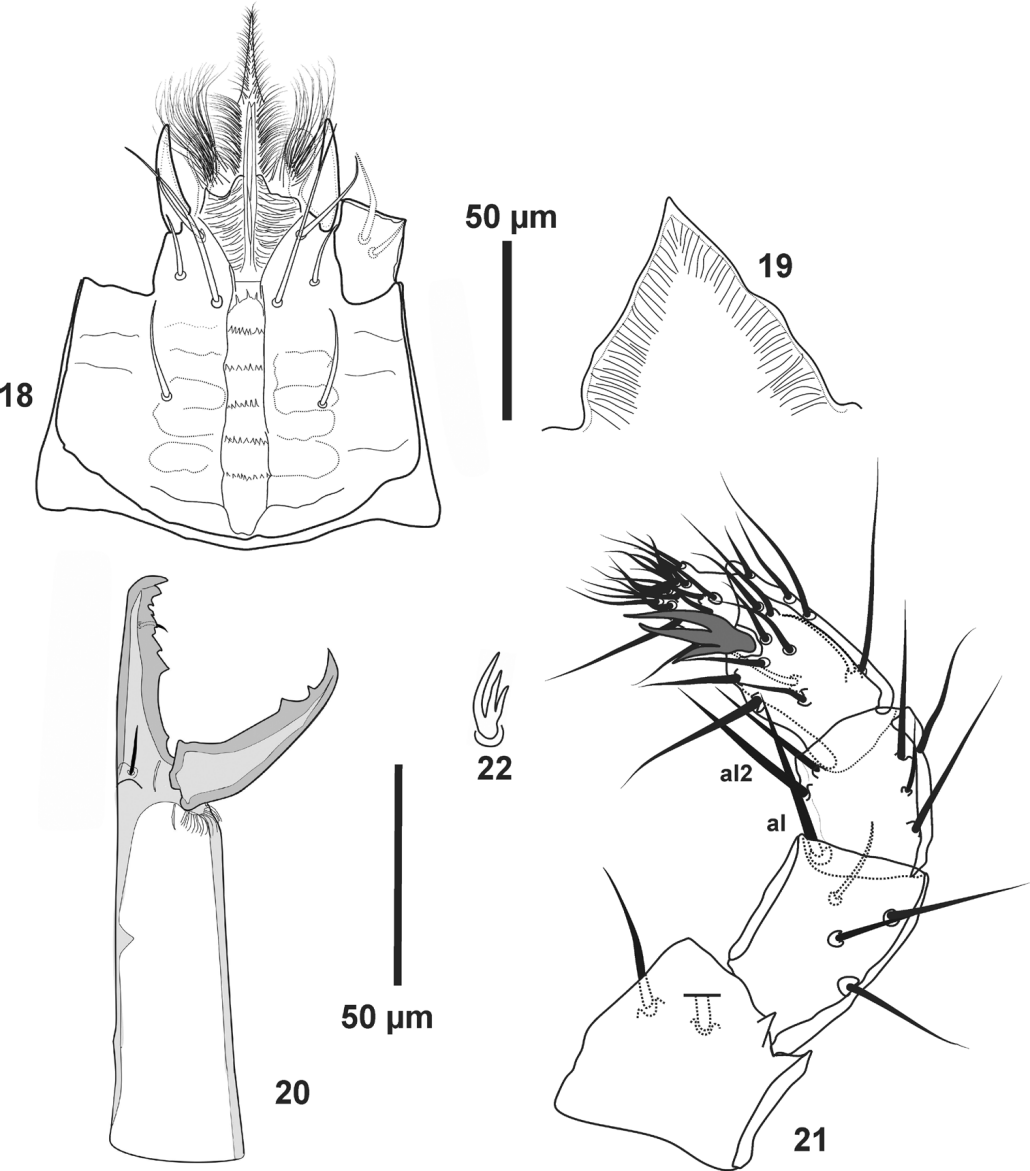
*Pogonolaelaps beaulieui* Nemati & Gwiazdowicz, gen. n., sp. n. (Female): **18** Hypostome **19** Epistome **20** Chelicera **21** Palp **22** Apotele.

***Gnathosoma*.** Deutosternal groove with six rows of denticles, corniculi horn-like, internal malae free medially and densely fringed, in addition possess two detachments of densely and very elongate hairs at basal part of each internal mala; labrum elongate, densely pubescent (Figs 7, 13, 18). Epistome sub-triangular with smooth antero-lateral margins (Figs 8, 19). Chelicerae chelate-dentate with prominent dorsal seta, lyrifissure, arthrodial processes and moderately robust setaceous pilus dentilis, movable digit with two teeth (Figs 9, 20). Digit-like male spermatodactyl simple and free distally (Figs 14, 28). Palp chaetotaxy normal (sensu Evans and Till 1965), numbers of setae on palp trochanter-tarsus: 2, 5, 6, 14 and 15 with aciculate and smooth setae, except *al1-2* of palp genu aciculate and slightly thickened; palp-tarsal apotele three-tined, basal tine smaller (Figs 21–22).

**Figures 23–26.**
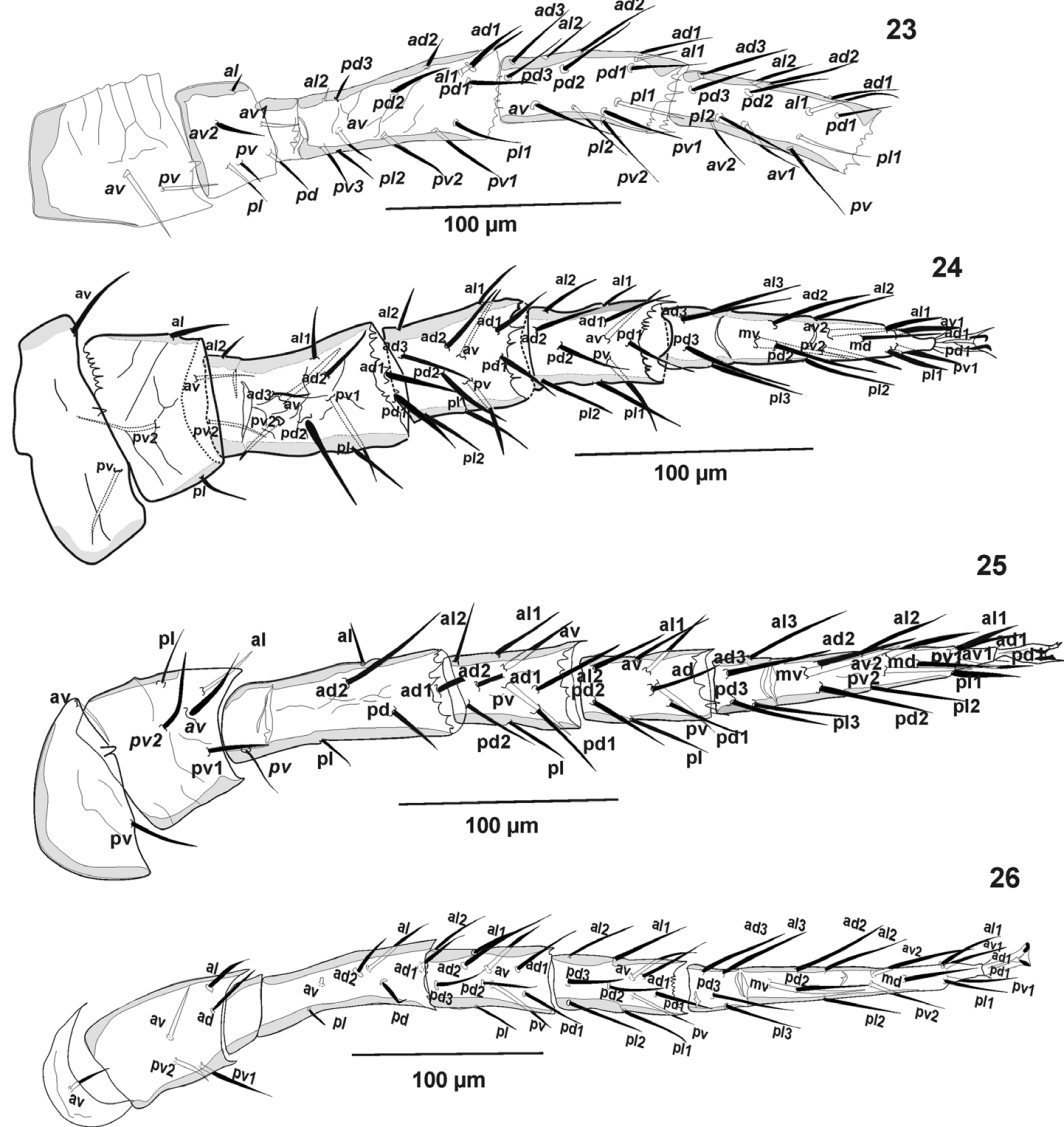
*Pogonolaelaps beaulieui* Nemati & Gwiazdowicz, gen. n., sp. n. (Female): **23** Leg I **24** Leg II **25** Leg III **26** Leg IV.

***Legs*.** Tarsi I–IV with claws and ambulacra (Figs 1, 10, 23–26). Leg chaetotaxy as follows: **leg I:** (Fig. 23) coxa 0 0/1 0/1 0, trochanter 1 1/2 0/1 1, femur 2 3/2 2/2 2, genu 2 3/2 3/1 2, tibia 2 3/2 3/1 2. **Leg II:** (Fig. 24) coxa 0 0/1 0/1 0, trochanter 1 0/1 0/2 1, femur 2 3/1 2/2 1, genu 2 3/1 2/1 2, tibia 2 2/1 2/1 2, tarsus 3,3/2,3/2,3 + *mv*, *md.* **Leg III:** (Fig. 25) coxa 0 0/1 0/1 0, trochanter 1 0/1 0/2 1, femur 1 2/0 1/1 1, genu 2 2/1 2/ 1 1, tibia 2 1/1 2/1 1, tarsus 3 3/2 3/2 3 + *mv*, *md.* **Leg IV:** (Fig. 26) coxa 0 0/1 0/0 0, trochanter 1 0/1 0/2 1, femur 0 2/1 1/1 1, genu 2 2/1 3/1 1 (Figs 6, 10, 26), tibia 2 1/1 3/1 2, tarsus 3 3/2 3/2 3 + *mv*, *md*.

**Figures 27–29.**
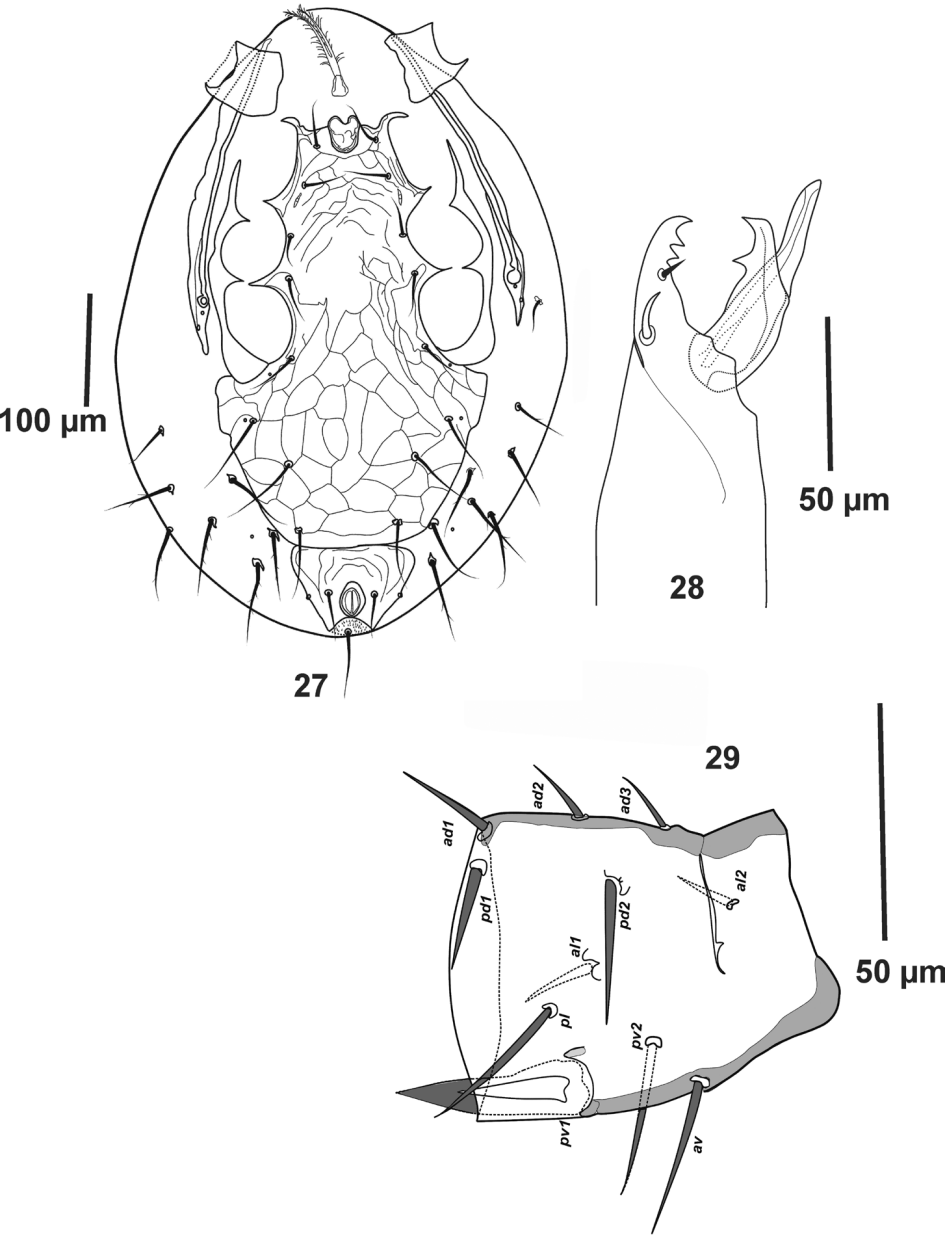
*Pogonolaelaps beaulieui* Nemati & Gwiazdowicz gen. n., sp. n. (Male): **27** Ventral idiosoma **28** Chelicera **29** Femur II (lateral view).

#### Etymology.

The name of the new genus is derived from the Greek word *pogon* (beard), and refers to the unusually long internal malae (gender masculine).

#### Note on subfamily placement of new genus.

Evans and Till (1966) proposed six subfamilies for laelapid mites. We consider our new genus as a member of Laelapinae subfamily (*sensu* Evans and Till 1966) based on the presence of attributes of the subfamily: presternal area in the female reticulated or with pre-endopodal shields, deutosternum with five to seven (usually six) transverse rows of denticles, chelicerae in the female chelate-dentate or rarely chelate-edentate, pilus dentilis present, in the male chelate-dentate with spermadactyl free anteriorly or partially fused with the movable digit, hypertrichy of dorsal shield when present usually restricted to the region of the *J* series of setae, male with holoventral shield or with discrete sternito-genito-ventral and anal shields.

This subfamily contains seven genera: *Ololaelaps*, *Androlaelaps*, *Ondatralaelaps*, *Laelaps*, *Hyperlaelaps*, *Pseudoparasitus* and *Hypoaspis* sensu lato with nine subgenera for the latter like: Hypoaspis (Hypoaspis), Hypoaspis (Alloparasitus), Hypoaspis (Stratiolaelaps), Hypoaspis (Cosmolaelaps), Hypoaspis (Penumolaelaps), Hypoaspis (Gymnolaelaps), Hypoaspis (Holostaspis), Hypoaspis (Laelaspis) and Hypoaspis (Gaeolaelaps), which are considered as full generic status presently (Trägårdh 1952, Hunter 1966, Lindquist et al. 2009, Rosario 1981, Beaulieu 2009, Joharchi et al. 2011).

#### Remarks.

Members of *Pogonolaelaps* gen. n. in general appearance may be similar to those genera of Laelapinae *sensu* Evans & Till (1966), which possess a broad epigynal shield such as *Laelaspis*, *Gymnolaelaps*, *Pseudoparasitus*, and due to the absence of *st4* and presence of hypertrichy on dorsal shield to *Laelaspisella* (was considered in this subfamily by Marais and Loots 1969) and for its special shape of dorsal setae with small knob at their base to *Cosmolaelaps* genera.

*Pogonolaelaps* may be similar to *Laelaspis* but it can be distinguished readily by the following characters. In *Laelaspis* palptarsal claw is two-tined (three tined in *Pogonolaelaps*); genital shield in *Laelaspis* with characteristic ornamentation including two distinct Λ-shaped lines; with two pairs of setae on extreme edges of shield in addition to *st*5 (in *Pogonolaelaps* without this special ornamentation and with only one pair of setae on the shield margin); *Laelaspis* usually lacks pre-sternal shield (present in *Pogonolaelaps*, but not markedly sclerotized). *Laelaspis* genu IV with nine setae including one ventral seta (*Pogonolaelaps* with ten setae including two ventral setae: *av* and *pv*); male of *Laelaspis* with holoventral shield (*Pogonolaelaps* with separate sub-triangular anal shield); dorsal shield in *Laelaspis* lacks hypertrichy (*Pogonolaelaps* with hypertrichy on opisthonotal part); *Laelaspis* members with *st4* and pore-like *iv3* on integument posterior to sternal shield (*Pogonolaelaps* lacks *st4* and *iv3* located on posterolateral extension of sternal shield near *st3*).

*Pseudoparasitus* and *Gymnolaelaps* members have *st4* and pore-like *iv3* on integument posterior to sternal shield, conspicuous denticulate epistome, genu IV with one ventral seta (*av*), and holotrichous dorsal shield with acicular setae; known males have holoventral shield and lack large spine or spur-like setae on femur II. *Pseudoparasitus* has large genital shield with 4–5 pairs of setae including two pairs on the shield surface; however, all of those in *Gymnolaelaps* are located on the lateral margin of shield. *Pogonolaelaps* lacks *st4* and *iv3* located on posterolateral extension of sternal shield near *st3*, with smooth sub-triangular epistome, large genital shield with only one pair of setae (*st5*) on the lateral margin, genu IV with two ventral seta (*av* and *pv*), dorsal shield possesses setae with small knob at their base, opisthonotal part with hypertrichous condition, males have separate anal shield and have large spine-like seta (*pv*) on femur II.

*Laelaspisella* (*Laelaspisella macrodorsalis* and *Laelaspisella epigynialis*) (Marais and Loots 1969) has dorsal shield hypertrichous for both the podonotal and opisthonotal parts, and with very small acicular setae, setae *Jv5* and *Zv5* are spatulate and pilose, chelicera lacks dorsal seta, the setation of genu I is deficient in one postero-dorsal seta (2 3/2 2/1 2), while *Pogonolaelaps* has dorsal setae with small knob at their base, hypertrichous condition only of opisthonotal region, setae *Jv5* and *Zv5* acicular, chelicerae possess dorsal setae; the setation of genu I is typical with three *pd* setae (2 3/2 3/1 2). Differences among *Pogonolaelaps* gen. n. and other related genera such as *Laelaspis*, *Gymnolaelaps*, *Pseudoparasitus*, *Laelaspisella* and *Cosmolaelaps* are mentioned in Table 1.

**Table 1.** Comparison among *Pogonolaelaps* gen. n. and other related laelapid genera.

	*Laelaspis*	*Gymnolaelaps*	*Pseudoparasitus*	*Laelaspisella*	*Cosmolaelaps*	*Pogonolaelaps*
**Type species**	*Laelaps astronomicus* Koch,1839	*Laelaps myrmecophilus* Berlese, 1892	*Laelaps meridionalis* G & R Canestrini, 1882	*Laelaspisella epigynialis* Marais & Loots, 1969	*Laelaps claviger* Berlese, 1883	*Laelaps canestrinii* Berlese, 1903
**Palp tarsal claw**	two-tined	three tined, third tine occasionally reduced, rarely absent	three-tined, posterior tine small	two-tined	two-tined	three-tined
**Two distinct Λ-shaped lines on genital shield**	present	absent	absent	absent	absent	absent
**Posterior margin of genital shield**	rounded	straight	straight	nearly rounded or tapered	usually rounded	rounded
**Setae on genital shield**	all on edges of shield	all on edges of shield	at least two pairs well inside edges of shield	with only *st5* on the margin of shield	with only *st5* on the margin of shield	all on edges of shield
**Pre-sternal plates**	absent	present	present	present	usually present	present and not so discernable
**Podal plates behind coxae IV**	present	usually present	present	medium size, rounded	absent	medium size, rounded
**Epistome**	nearly always smooth	denticulate	denticulate	smooth	denticulate	smooth
**Hypertrichy of dorsal shield**	absent	absent	absent	present on whole dorsum	absent	present only on opisthonotal part
**Dorsal shield setae**	nearly always scimitar-shaped with small knob at their bases	acicular	acicular	acicular	with different shaped but never acicular	with small knob at their bases
**Setae *av* and *pv* on genu IV**	only with *av*	only with *av*	only with *av*	with *av* and *pv*	only with *av*	with *av* and *pv*
**Seta *pd3* on tibia I**	present	present	present	absent	present	present
**Male holoventral shield**	present	present	present	?	nearly always present	absent, with separate sub-triangular anal shield
**Large spine or spur-like seta(e) on male leg II**	absent	absent	absent	?	absent	present
**Seta *st4***	present	present	present	absent	present	absent
**Spatulate elongate *Jv5* and *Zv5***	absent	absent	absent	present	absent	absent
**Cheliceral dorsal seta**	present	present	present	absent	present	present
**Internal malae with elongate densely hairs**	absent	absent	absent	present	absent	present

### Pogonolaelaps canestrinii

Taxon classificationAnimaliaMesostigmataLaelapidae

(Berlese, 1903) comb. n.

Figures 1–6 , 7–10 , 11–14

Laelaps canestrinii Berlese, 1892: LXIX, 1.
Laelaps (Eulaelaps) canestrinii Berlese, 1903: 13.
Hypoaspis canestrinii . — Oudemans 1902: 24; Oudemans 1903; 129; Buitendijk 1945: 295.
Laelaspis (Hypoaspis) canestrinii . — Berlese 1904.
Gymnolaelaps canestrinii . — Vitzthum 1929: 25; Sellnick 1931: 695; Costa 1962: 491: Costa 1966: 74; Bhattacharyya 1968: 539; Joharchi et al. 2011: 23.
Laelaps canestrinii . — Tipton 1960: 290.
Hypoaspis (Gymnolaelaps) canestrinii . — Hunter 1967: 99; Bregetova 1977: 523.
Pseudoparasitus (Gymnolaelaps) canestrinii . — Karg 1981: 218; Karg 1989, 334; Karg 1993: 135.
Hypoaspis (Cosmolaelaps) canestrinii . — Karg 1979: 71.
Laelaspisella canestrinii . — Joharchi and Halliday 2013: 46; Moreira 2014: 316.

#### Specimens examined.

Khuzestan province: Ahwaz (31°19'22"N, 48°40'50"E, H: 16 m), nest of unknown ant, two females, coll. A. Nemati, 2012; Baghmalek (31°31'22"N, 49°53'8"E, H: 707m), soil, one female and one male, coll. A. Nemati, 2012; Ghaletol (31°37'55"N, 49°53'20"E, H: 885 m), soil and nest materials of unknown ant, one female, coll. A. Nemati, 2012; Izeh (31°49'52"N, 49°52'9"E, H: 845 m), soil, two females, coll. A. Nemati, 2012 and Masjed-Soleiman (31°56'11"N, 49°18'14"E, H: 251 m), soil, one female, coll. A. Nemati, 2011. Chaharmahal Va Bakhtiari province: Shahrekord (32°19'39"N, 50°51'35"E, H: 2206 m), soil, three females, coll. A. Nemati, 2009, Lordegan (31°30'30"N, 50°49'39"E, H: 1594 m), soil, three females and two males, coll. A. Nemati, 2012; Naghan (31°56'19"N, 50°44'54"E, H: 2219 m), soil, one female, coll. M. Mohseni, 2010. Ben (32°32'32"N, 50°43'48"E, H: 2203 m), soil, four females and one male, coll. A. Nemati, 2011. Esfahan province: Esfahan (32°39'37"N, 51°41'22"E, H: 1608 m), soil, coll. A. Nemati, 2011. Kashan, soil, five females, coll. M. Fahiminezhad, 2006. Shahreza, soil, one female (32°07'N, 51°55'E, alt. 1725 m), 22 August 2010; one female (32°01'N, 51°53'E, alt. 1800 m), 20 March 2011; one female (32°01'N, 51°53'E, alt. 1806 m), 4 April 2011; three females (32°02'N, 51°51'E, alt. 1827 m), 11 June 2011; five females and two males (31°39'N, 51°55'E, alt. 2220 m), 9 July 2011; one female and one male (32°00'20"N, 51°52'54"E, alt. 1823 m), 17 July 2011; two females (31°56'N, 51°44'E, alt. 1963 m), 4 August 2011. Microslides were deposited in APAS.

Explanation concerning Berlese’ specimens were cited in the following text under notes on the male of *Pogonolaelaps canestrinii*.

#### Diagnosis.

Podonotal region with 23 pairs of thin small setae with small knobs at their base (except for *j1* and *z1*); opisthonotal region with 32 pairs of setae, three unpaired setae between *J* series and seven pairs of long barbed setae; pre-sternal area with a pair of indistinct poorly sclerotized plates; *iv1-3* slit-like and located on the sternal shield surface; peritremes long, extending to coxa I anteriorly; internal malae densely fringed. Epistome with smooth anterior margin. Palp apotele three-tined; genu IV with 10 setae, including two ventral setae (*av* and *pv*).

#### Description of the female

(n = 7). Figures 1–10.

***Dorsal idiosoma*.** Dorsal shield oval-shaped, length 624–723, width at level of setae *r3* 425-465; reticulation more distinct on opisthonotal part; podonotal region with 23 pairs of thin small setae with small knobs at their base (except for *j1* and *z1*); opisthonotal region with 32 pairs of setae, three unpaired setae between *J* series and seven pairs of long barbed setae (Fig. 5). Dorsal setae short (26–36) except for longer setae on opisthonotal part (52–83). Dorsal idiosoma with 19 pairs of lyrifissures and pore-like structures.

***Ventral idiosoma*** (Figs 1–4). Tritosternum with tubular base (39–47) and pilose laciniae (65–80). Pre-sternal area with a pair of indistinct poorly sclerotized plates (Figs 1, 2). Sternal shield (Figs 1, 2) reticulate anteriorly and laterally, smooth posteriorly, 80–96 long, 107–122 wide, anterior margin sinuate, convex medially, posterior margin deeply concave, sternal setae smooth, *st1* (46–67), *st2* (47–50), *st3* (44–57), *iv1* slit-like, located slightly anterior to *st1*; *iv2* slit-like, between *st2-st3*, *iv3* slit-like, located on postero-lateral corners of sternal shield; *st4* absent. Genital shield (Figs 1, 3) broad, well ornamented, 346–374 long (including hyaline flap at the base of posterior margin of sternal shield), 177–195 wide at level of *st5* and widest (239–260) near setae *Zv1*, abutting anal shield, bearing one pair of setae (*st5* 44–49); paragenital pores (*iv5*) on soft integument posterior to genital setae. Anal shield (Fig. 4) sub-triangular, reticulated, 99–109 long, 177–195 wide, post-anal seta (43–45) slightly longer than para-anal setae (36–42). Cribrum extending laterally to level of post-anal seta. Opisthogastric surface with: one pair of elongate metapodal plates plus two pairs of platelets (Figs 1, 3); 10–11 pairs of setae, 36–49 μm long; and seven pairs of pore-like structures, plus one pair (*gv3*) on lateral margin of anal shield. Stigmata surrounded by narrow, pointed stigmatal plate, which extends posteriorly past level of mid-coxae IV. Peritremes long, extending to anterior of coxa I. Endopodal, podal and exopodal plates as in genus.

***Gnathosoma*.** Hypostome (Fig. 7) with three pairs of similar smooth simple setae (*h1*:69–79, *h2*: 21–30, *h3*: 72–84) plus one pair of palpcoxal setae (40–47). Deutosternal groove with six rows of denticles; corniculi normal, horn-like, reaching mid-level of palp femur; internal malae densely fringed with very elongate hairs. Epistome (Fig. 8) with smooth anterior margin. Arthrodial processes of chelicerae (Fig. 9) developed, movable digit (47–49) with two teeth, middle article (174–187), fixed digit with six teeth, setaceous pilus dentilis small. Palp attributes as in the genus.

***Legs*.** Tarsi I–IV with claws and ambulacra. Legs I (559–580) and IV (587–605), longer than legs II (429–450) and III (429–455). Genu IV (Figs 6, 10) with 10 setae, including two ventral setae (*av* and *pv*).

#### Description of the male

(n = 5). Figures 11–14.

***Dorsal idiosoma*** (Fig. 11). Dorsal shield 512–540 long, 300–315 wide, dorsal chaetotaxy as for female, except setae that are slightly shorter.

***Ventral idiosoma*** (Fig. 12). Presternal area with transverse lines, not well sclerotised; sternito-genito-ventral shield, 351–357 long, 213–234 wide, reticulated throughout, bearing eight pairs of simple pointed setae, *st1*-*st5* (26–36), *Zv1* and *Jv1–2* (36–45); with separate reticulated anal shield, 60–75 long, 94–106 wide; without metapodal plates. Soft cuticle with ten pairs of pointed, barbed setae.

***Gnathosoma*.** As in female (Fig. 13); chelicerae (Fig. 14) with middle segment (122–130), fixed digit (34–36) bearing two teeth. Pilus dentilis setiform. Movable digit (34) with one tooth; spermatodactyl (41–44).

***Legs*.** Tarsi I-IV with claws and ambulacra. leg I (490–556), leg II (354–400), leg III (387–411), leg IV (569–577), legs I and IV longer than legs II and III. Structure and chaetotaxy as in female, except femur II, which bears a spine-like *pv1* seta.

#### Notes on the male of *Pogonolaelaps canestrinii* (Berlese), comb. n.

The described males of *Gymnolaelaps* have a holoventral shield. Some confusion about the state of the male ventral shields in *Pogonolaelaps canestrinii* (Berlese), comb. n. exist as Berlese (1892) originally showed the anal shield not separated, but later Berlese (1904) illustrated the species with a separate anal shield. Costa (1962) and Hunter (1967) regarded the species as having a separate anal shield, and here we confirm this by checking the type specimens of *Pogonolaelaps canestrinii* comb. n. kindly examined by Dr. Roberto Nanelli, and the type information is as follows:

Slide 4 Myrm./45 labeled *Laelaps canestrinii* Berl., 1 female, type, nidi formiche, Portici; (nests of ant, Portici: a locality near the city of Naples, Italy); Slide 4 Myrm./46 labelled *Laelaps canestrinii* Berl., 1 female, nidi formiche, Portici; Slide 4 Myrm./47 labelled *Laelaps canestrinii* Berl., 3 females, type, nidi formiche, Portici; Slide 4 Myrm./48 labelled *Laelaps canestrinii* Berl., 1 male, type, nidi formiche, (without locality of collection) (perhaps Portici); Slide 221/31 labelled Hypoaspis (Gymnolaelaps) canestrinii Berl., female, Spalato, libero nell’humus; (free, vacant in humus); The specimens are in poor condition but it is possible to see that the female’s dorsal shield has short setae, plus seven long thickened posterior setae, very similar to that shown in Figures 1, 11, 12. The slides labelled Myrm.= associated with ant, myrmecophilous.

Berlese (1892) described female and male specimens of *Laelaps canestrinii*, and according to his figures the female possesses a very wide epigynal shield with four pairs of setae in addition to *st5* and with straight posterior margin, lacks setae between the epigynal and anal shields, sternal shield only with two pairs of setae, lacks the metasternal plates and setae, the movable digit of the chelicera with three teeth, and male without separate anal shield. Berlese (1904) redescribed Laelaps (Hypoaspis) canestrinii as epigynal shield of the female posteriorly rounded, carries only *st5*, possess one pair of setae between the epigynal and anal shields. In the male, the anal shield is clearly separate from the genito-ventral shield. The examination of the type material of *Pogonolaelaps canestrinii* and figures by Berlese (1904) confirm the identity of specimens redescribed in this study.

### Pogonolaelaps beaulieui gen. n., sp. n.

Taxon classificationAnimaliaMesostigmataLaelapidae

http://zoobank.org/F2A5A9D9-2409-4AF1-ACE7-3572A4463DAF

Figures 15–17 , 18–22 , 23–26 , 27–29

#### Specimens examined.

Holotype, female, Chaharmahal va Bakhtiari province, Shahrekord (32°19'39"N, 50°51'35"E, H: 2206 m), soil, 2009; coll. A. Nemati; Chaharmahal va Bakhtiari province, Saamaan, Cham-Khalifeh (32°30'35"N, 50°52'12"E, H: 1875 m), Walnut rooting wood, 13 June 2012, three females, coll. A. Nemati; Saamaan (32°30'36"N, 50°53'13"E, H: 1873 m), rooting wood, three females and two males, 27 May 2012, coll. A. Nemati; Shahrekord, Shahrekord University, nest materials of unknown ant, one female and one male, 31 August 2007, coll. A. Nemati; Shahrekord, Shahrekord University, nest materials of unknown ant, two females, August 2006, coll. H. Maleki.

#### Diagnosis.

*iv3* slit-like located posterior to *st3* on postero-lateral corners of sternal shield, with large crescent-shaped podal shield posterior to coxa IV; dorsal shield with 23 pairs of setae on podonotal (*rx* seta present on podonotal part), and 28 pairs on opisthonotal part, plus 1-3 unpaired setae between *J* series; 7–9 pairs of thickened, elongated, and barbed opisthonotal setae; genu I with normal chaetotaxy (2 3/2 3/1 2), and genu IV with ten setae including two ventral setae (*av* and *pv*).

#### Description of the female

(n = 7). Figures 15–26.

***Dorsal idiosoma*** (Fig. 15). Dorsal shield length 728–780, width 517–560, oval shaped, wraps around and flaps onto the ventral idiosoma; reticulation more distinct on opisthonotal part, with 51 pairs of setae, 23 pairs on podonotal, 28 pairs on opisthonotal region, plus 1–3 *Jx* setae between *J* series (usually with three); setae increasing in length from anterior to posterior and from dorso-central to dorso-lateral part, dorso-central setae length on podonotal (23–42) and dorso-lateral setae (50–80), lateral opisthonotal setae tend to reach well past base of next posterior setae, lateral opisthonotal part with 7–9 pairs of long, thick, barbed setae (98–130), length of central opisthonotal setae 31–52, lateral opisthonotal setae 62–75. Dorsal setae scimitar-shaped with a small basal knob (Fig. 17). Dorsal shield with 19 pairs of pore-like structures, nine pairs on podonotal and ten pairs on opisthonotal (six pairs of those are large and slit-like) (Fig. 15).

***Ventral idiosoma*** (Fig. 16a, b). Tritosternum with columnar base (19–26) and paired pilose laciniae (85–93); pre-sternal plates weakly sclerotized and ornamented with transverse lines. Sternal shield with reticulate anterior and lateral margins, smooth posteriorly, 86–94 long, narrowest between coxae II (146–156), widest between coxae II and III (213–221), anterior margin sinuate, convex medially, posterior margin deeply concave. Sternal setae smooth, *st1* (75–83), *st2* (62–70) and *st3* (62–78), *iv1* slit-like, located slightly anterior to *st1*; *iv2* slit-like, between *st2* and *st3*, *iv3* slit-like located posterior to *st3*, on postero-lateral corners of sternal shield; *st4* absent. Endopodal plates II/III fused to lateral margins of sternal shield, endopodal plates III/IV elongate, curved. Genital shield broad, 377–395 long (including hyaline flap at the base of posterior margin of sternal shield), 208–226 wide at level of *st5* and widest (260–273) near setae *Zv1*, abutting anal shield, well ornamented, with one pair of setae (*st5* = 73–75) on shield and three pairs of setae adjacent to lateral edges; circular paragenital pores (*iv5*) located on soft integument between coxa IV and pair of minute narrow platelets. Anal shield subtriangular, 125–133 long, 151–156 wide, reticulated, post-anal seta (65–75) slightly longer than para-anal setae (52–60). Cribrum in a strip form of teeth, extending antero-laterally to level of post-anal seta. Opisthogastric surface with: one pair of elongate metapodal plates, two pairs of minute platelets (Fig. 16a), 12 pairs of long setae (Figs 16a,b) (*Jv1* 91–93, *Jv2* 83–88, *Jv3* 78–86, *Jv4–5* 98–104, *Zv1* 96–99, *Zv2*–3 88, *Zv4*–5 98–104), and four pairs of pore-like structures, plus one pair of adanal gland pores *gv3* on lateral margin of anal shield (Figs 16a, b). Endopodal, podal and exopodal plates are as in genus. Peritreme extending from coxa IV to anterior level of coxa I, peritrematal shield wide, with two pairs of post-stigmatal pores, one pair of small pores anterior to stigmata and two pairs of pores (*ip*, *gp*) at level of coxae II/III.

***Gnathosoma*.** Hypostome with three pairs of smooth simple setae: *h1*, *h3* (70–75), *h2* (23–26). Palpcoxal setae 36–39. Deutosternal groove with six rows of denticles, each bearing 7–9 small teeth except first row with three larger teeth; corniculi normal, horn-like, reaching beyond of mid-level of palp femur; internal malae free medially and densely fringed with elongate threads (Fig. 18). Epistome sub-triangular with smooth antero-lateral margins (Fig. 19). Chelicera with dorsal seta, lyrifissure and developed arthrodial processes (Fig. 20), movable digit (44) with two teeth, middle article (182–190), fixed digit with two moderately large teeth proximal to pilus dentilis, followed by four minute teeth and a small offset tooth subapically, setaceous pilus dentilis moderately robust. Palp chaetotaxy normal (*sensu* Evans and Till 1965) and as in genus, with simple setae except *al* on femur, and *al2* of genu slightly thickened (Fig. 21), palp apotele three-tined (Figs 21, 22).

***Legs*.** Tarsi I–IV with claws and ambulacra. Legs I (595–647) and IV (699–704), longer than legs II (455–486) and III (478–509) (excluding pre-tarsus). Leg chaetotaxy as follows: **leg I:** (Fig. 23) coxa 0 0/1 0/1 0, trochanter 1 1/2 0/1 1, femur 2 3/2 2/2 2, genu 2 3/2 3/1 2, tibia 2 3/2 3/1 2. **Leg II:** (Fig. 24) coxa 0 0/1 0/1 0, trochanter 1 0/1 0/2 1, femur 2 3/1 2/2 1, genu 2 3/1 2/1 2, tibia 2 2/1 2/1 2, tarsus 3,3/2,3/2,3 + *mv*, *md.* **Leg III:** (Fig. 25) coxa 0 0/1 0/1 0, trochanter 1 0/1 0/2 1, femur 1 2/0 1/1 1, genu 2 2/1 2/ 1 1, tibia 2 1/1 2/1 1, tarsus 3 3/2 3/2 3 + *mv*, *md*. **Leg IV:** (Fig. 26) coxa 0 0/1 0/0 0, trochanter 1 0/1 0/2 1, femur 0 2/1 1/1 1, genu 2 2/1 3/1 1, tibia 2 1/1 3/1 2, tarsus 3 3/2 3/2 3 + *mv*, *md*.

***Insemination structures*.** Not seen, apparently unsclerotised.

#### Description of the male

(n = 3). Figures 27–29.

***Dorsal idiosoma*.** Dorsal shield length 585–606 long, width 457–470, dorsal chaetotaxy as for female, except setae which are slightly shorter.

***Ventral idiosoma*** (Fig. 27). With weakly sclerotised presternal shields; sterniti-genito-ventral shield, 413–420 long, 247–257 wide, reticulated throughout, bearing eight pairs of simple, pointed setae, *st1*-*st5* (38–42), *Zv1* and *Jv1–2* (58–63); with separate reticulated anal shield, 99–105 long, 110–122 wide, post-anal seta (55–59) longer than para-anals (40–44); without metapodal plates, apparently fused to the lateral margin of sterniti-genito-ventral shield. Soft cuticle with eight pairs of pointed, mostly barbed setae.

***Gnathosoma*.** As in female; chelicerae (Fig. 28) with middle segment (133–141), fixed digit (38–40) bearing two teeth. Pilus dentilis setiform. Movable digit (35–37) with one tooth; spermatodactyl (45) relatively straight.

***Legs*.** Tarsi I-IV with claws and ambulacra. Leg I (510–525), leg II (390–401), leg III (438–445), leg IV (582–603), legs I and IV longer than legs II and III. Structure and chaetotaxy as in female, except for femur II, which bears a spine-like and thickened *pv1* seta (Fig. 29).

#### Etymology.

This species is named in honor of Dr. Frederic Beaulieu (Canadian National Collection of Insects, Arachnids and Nematodes, Agriculture and Agri-Food Canada, Ottawa, Canada).

#### Remarks.

*Pogonolaelaps beaulieui* gen. n., sp. n. is similar to *Pogonolaelaps canestrinii* comb. n. and can be readily distinguished from it by the presence of 28 pairs of setae on opisthonotal region plus 1–3 unpaired setae (32 pairs in *Pogonolaelaps canestrinii* plus 3 unpaired setae between *J* series); the dorsal setae of *Pogonolaelaps beaulieui* gen. n., sp. n. are much longer than those of *Pogonolaelaps canestrinii* comb. n. (see text), and the genital shield in *Pogonolaelaps beaulieui* gen. n., sp. n. [377–395 long, 208–226 wide at level of *st5* and widest (260–273) near setae *Zv1*] is longer and wider than that of *Pogonolaelaps canestrinii* comb. n. [346-374 long, and 177-195 wide at level of *st5* and widest (239-260 μm) near setae *Zv1*].

## Discussion

Karg (1989) considered *Gymnolaelaps* as a subgenus of *Pseudoparasitus* based on the presence of a three-tined apotele, developed podal shields posterior to coxa IV, epistome with denticulate anterior margin and the presence of 1-3 pairs of setae on lateral edges of an expanded genital shield. Other authors defined *Gymnolaelaps* at the genus level, with diagnoses provided by Hunter (1967), Hunter and Costa (1971) and, most recently, Joharchi et al. 2011 and Joharchi and Halliday 2013. According to these last authors the genus is defined by: genu IV with nine setae including one ventral seta; the metasternal setae always present; dorsal shield covering dorsum and even extending ventrally, has a normal complement of 40 pairs of setae, often with paired *Zx* setae between *J* and *Z* setae, unpaired *Jx* setae also often present; dorsal shield setae distally pointed, smooth or slightly serrated, not long and whip-like; and *Zv1*, *Jv1-2* setae (additional to *st5*) are on the margin of the shield. The only character that separates *Gymnolaelaps* from *Pseudoparasitus* seems to be the given: in *Pseudoparasitus*, at least two pairs of ventro-genital setae are set well inside the edges of the shield (Joharchi et al. 2011, Joharchi and Halliday 2013).

The generic definition of *Gymnolaelaps* does not apply for all species that are assigned to this genus nowadays, as there are several characters not taken into account and excluded by the diagnosis of Joharchi et al. (2011) and Joharchi and Halliday (2013). These exceptions occur in the genito-ventral shield of female, the number of the apotele tines, the form of the anterior margin of the epistome, leg and dorsal chaetotaxy, and the form of the podal shield posterior to coxa IV.

*Gymnolaelaps shealsi* Hunter & Costa, 1971 has a genito-ventral shield that does not extend to the anal shied, lacks an expanded podal plate behind coxa IV and the epistome is triangular with smooth margins. *Gymnolaelaps viennensis* Sellnick is similar to *Gymnolaelaps shealsi* in the shape of genital shield. *Gymnolaelaps krantzi* (Hunter, 1967) has a two-tined apotele and the epistome has a smooth rounded anterior margin. *Gymnolaelaps unospinosus* (Karg, 1978) has thickened seta on femur II (not included in the diagnosis of Joharchi et al. 2011 and Joharchi and Halliday 2013) and has a very narrow podal plate behind coxa IV.

### Notes on *Pseudoparasitus reniculus* Karg, 1981 and *Pseudoparasitus triquetrus* Karg, 2003

Pseudoparasitus (Gymnolaelaps) reniculus Karg, 1981 has four pairs of setae on genital shield, of which *Jv1-2* inserted away from the shield margins and lacks the holotrichous condition on dorsal shield (slightly hypertrichous) on the opisthonotal part. According to our definition of *Gymnolaelaps* and the difference between *Gymnolaelaps* and *Pseudoparasitus* genera, we consider this species in its original genus *Pseudoparasitus* not in *Gymnolaelaps* as proposed by Joharchi et al. (2011) and Moreira (2014).

Pseudoparasitus (Gymnolaelaps) triquetrus was described by Karg (2003) in Ecuador, as a species in *Gymnolaelaps* (as subgenus) in which the genital shield expands behind coxa IV and *Zv1*, *Jv1-2* located on the surface of genital shield far from the edges. Based on these characters, it is also considered as a species in its original genus *Pseudoparasitus* and not in *Gymnolaelaps* as proposed by Moreira (2014).

### Notes on *Laelaspisella* Marais & Loots

*Laelaspisella* was originally described by Marais and Loots (1969) by discussing several morphological characters (Marais and Loots 1969). Joharchi and Halliday (2013) have defined *Laelaspisella* by considering three main characters: dorsal shield hypertrichy; absence of metasternal setae and genu IV bearing two ventral setae; however none of those are apomorphic characters for this genus. In addition to *Laelaspisella macrodorsalis* Marais & Loots, and *Laelaspisella epigynialis* Marais & Loots, *Gymnolaelaps canestrinii* and *Gymnolaelaps kabitae* were also transferred to *Laelaspisella* (Joharchi and Halliday 2013).

The new species here (*Pogonolaelaps beaulieui*), has hypertrichous dorsal shield (but in opisthonotal part), absent metasternal setae and genu IV with two ventral setae, which in accordance with the idea of Joharchi and Halliday (2013) puts it in genus *Laelaspisella*. However when considering some other characters within genus mentioned above, several problems would arise assigning this new species and *Pogonolaelaps canestrinii* to *Laelaspisella*. Herein we discussed it below.

First, *Laelaspisella* (*Laelaspisella macrodorsalis* and *Laelaspisella epigynialis*) (Marais and Loots 1969) has ovoid pre-endopodal plates reticulated and well sclerotised but in *Pogonolaelaps canestrinii* and *Pogonolaelaps beaulieui* gen. n., sp. n. the pre-endopodal plates are not so sclerotized. In *Gymnolaelaps* the posterior half of the pre-endopodal plate is usually strongly sclerotized, and the anterior half is less sclerotized. The pre-endopodal plates of *Laelaspisella macrodorsalis*, and *Laelaspisella epigynialis* are not described in sufficient detail, but according to the illustrations these ovoid plates are conspicuous and with line ornamentations.

Second, the sternal shield of *Laelaspisella* has two pairs of poroids or lyrifissures, *iv1* and *iv2*. The metasternal pores *iv3* apparently are absent (Marais and Loots 1969). *Pogonolaelaps canestrinii* comb. n. and *Pogonolaelaps beaulieui* gen. n., sp. n., have slit-like *iv3* on the surface of postero-lateral corners of sternal shield. However Marais and Loots didn’t mention about *iv3* in *Laelaspisella*, but according to their illustrations *iv3* are not present in *Laelaspisella macrodorsalis* and *Laelaspisella epigynialis*. Species of *Gymnolaelaps* have poroid *iv3* posterior to sternal shield on soft integument.

Third, Joharchi and Halliday (2013) have cited the absence of *st4* as one of the three main characters of *Laelaspisella* genus (*Laelaspisella macrodorsalis* and *Laelaspisella epigynialis*), but metasternal setae are also absent in some other taxa like genus *Reticulolaelaps* Costa, 1968 and some species of *Hypoaspis* *sens. lat.*, such as Hypoaspis (Hypoaspis) metapodalis Karg, 1978; Hypoaspis (Hypoaspis) eugenitalis Karg, 1978; Hypoaspis (Alloparasitus) pycnosis Karg, 1982 and Hypoaspis (Holostaspis) tridentata Karg, 1982 (These scientific names are mentioned here as in the related literature). This character could not be considered as apomorphic for *Laelaspisella* due to the presence in different genera and species. *Pogonolaelaps beaulieui* gen. n., sp. n. and *Pogonolaelaps canestrinii* comb. n. lack *st4*.

Fourth, the genito-ventral shield in *Laelaspisella* (*Laelaspisella macrodorsalis* and *Laelaspisella epigynialis*) (Marais and Loots 1969) is not reaching the anal shield and there are two pairs of setae between genito-ventral and anal shields. This shield has one pair of setae (*st5*), widened slightly behind the genital setae and is rounded or tapered posteriorly. This condition was observed in *Laelaspisella macrodorsalis* (posterior edge distinctly tapered), *Laelaspisella epigynialis* (posterior edge rounded or slightly tapered), but *Pogonolaelaps canestrinii* comb. n. and *Pogonolaelaps beaulieui* gen. n., sp. n. have genital shield broad, strongly widened posterior to genital setae, posterior edge rounded, and abutting anal shield. Such condition can be seen in some species of *Gymnolaelaps*.

Fifth, setae *Jv5* and *Zv5* are spatulate and pilose in *Laelaspisella* (*Laelaspisella macrodorsalis* and *Laelaspisella epigynialis*) (Marais and Loots 1969) and it is suspected to represent an apomorphic character. Setae *Jv5* and *Zv5* are acicular in *Pogonolaelaps canestrinii* comb. n., and *Pogonolaelaps beaulieui* gen. n., sp. n. (with small barbs), and all known species of *Gymnolaelaps*.

Sixth, in species of *Laelaspisella* (*Laelaspisella macrodorsalis* and *Laelaspisella epigynialis*), and some species of *Gymnolaelaps* like, *Gymnolaelaps krantzi* (Hunter) and *Gymnolaelaps obscuroides* (Costa) the palp apotele has two tines. In *Gymnolaelaps*, there is a variation of the palp apotele and most species have the apotele 3-tined, but in a few species the third tine is reduced or lost. Maybe this represents a secondary loss of the third tine in some species. In *Pogonolaelaps canestrinii*, and *Pogonolaelaps beaulieui* gen. n., sp. n. it is 3-tined.

Seventh, *Laelaspisella* species (*Laelaspisella macrodorsalis* and *Laelaspisella epigynialis*) lacks dorsal seta of chelicerae, but *Gymnolaelaps* members and also *Pogonolaelaps beaulieui* gen. n., sp. n. and *Pogonolaelaps canestrinii* have this seta.

Eighth, the anterior margin of the epistome is smooth and sharply mucronated in *Laelaspisella macrodorsalis* and *Laelaspisella epigynialis*. In *Pogonolaelaps canestrinii*, and *Pogonolaelaps beaulieui* gen. n., sp. n. the anterior margin is smooth and pointed but not as sharply mucronate as in former. The most species of *Gymnolaelaps* have denticulate epistome.

Ninth, species of *Laelaspisella* (*Laelaspisella macrodorsalis* and *Laelaspisella epigynialis*) have the dorsal shield hypertrichous in both the podonotal and opisthonotal region with very small and acicular setae. Hypertrichous condition can be seen in the other laelapid mites like genus *Reticulolaelaps* (on whole dorsal shield), some species of *Pneumolaelaps*, and *Gaeolaelaps ciconia* (Karg, 1982): with this character in opisthonotal and opisthogastric regions, *Gaeolaelaps ardoris* (Karg, 1993): on both podonotal and opisthonotal parts. In *Pogonolaelaps canestrinii*, and *Pogonolaelaps beaulieui* gen. n., sp. n. dorsal shield is hypertrichous only in the opisthonotal region and dorsal setae possess small knob at their base. The podonotal region with holotrichous situation and *rx* located on the shield. *Gymnolaelaps* species have holotrichous condition with acicular setae pointed distally, and lack *rx* on the shield.

Tenth, in *Laelaspisella* species (*Laelaspisella macrodorsalis* and *Laelaspisella epigynialis*) the setation of genu I is deficient in one postero-dorsal seta (2 3/2 2/1 2). According to the Evans and Till (1965) and Lee (1970) it can be stated that the chaetotaxy of genu I is more stable than genu IV in mesostigmatid mites. Evans and Till (1965) noted some exceptions to the normal chaetotaxy of genu I (2 3/2 3/1 2) in some taxa of Dermanyssoidea such as *Pseudolaelaps doderoi*, *Dermanyssus* spp. and *Ornithonyssus* spp., that have been excluded from Laelapidae and at present are members of Pachylaelapidae, Dermanyssidae and Macronyssidae, respectively (Evans and Till 1965, Lindquist et al. 2009). For this reason, genu I in laelapid mites has normal chaetotaxy. *Laelaspisella* (*Laelaspisella macrodorsalis* and *Laelaspisella epigynialis*) has unique chaetotaxy in genu I with only two postero-dorsal setae (2 3/2 2/1 2), so this can be considered as an apomorphic character for *Laelaspisella* (*Laelaspisella macrodorsalis* and *Laelaspisella epigynialis*). *Gymnolaelaps* species, as most other free-living laelapids, have three postero-dorsal setae (2 3/2 3/1 2) on genu I. *Pogonolaelaps beaulieui* gen. n., sp. n. and *Pogonolaelaps canestrinii* are no exception with a typical set of setae.

Eleventh, in *Laelaspisella* species (*Laelaspisella macrodorsalis* and *Laelaspisella epigynialis*) the chaetotaxy of genu IV deviates from the normal (2 2/1 3/0 1) due to the presence of a postero-ventral seta (2 2/1 3/1 1).

Joharchi and Halliday (2013) emphasized the presence of two ventral setae on genu IV as one of the three main characters of *Laelaspisella* species (*Laelaspisella macrodorsalis* and *Laelaspisella epigynialis*). The greatest diversity of leg segments chaetotaxy in laelapid mites has been observed in genu IV. In addition to normal pattern (2 2/1 3/0 1), three other types of chaetotaxy have been observed (Evans and Till 1965): in *Laelaps agilis* (2 2/1 3/1 2); *Laelaspisella echidninus*, *Laelaspisella hilaris*, *Laelaspisella muris*, *Hyperlaelaps amphibian*, *Hyperlaelaps microti*, *Eulaelaps stabularis*, *Eulaelaps nova*, *Haemolaelaps casalis*, *Haemolaelaps fahrenholzi*, *Euandrolaelaps sardoa* and *Laelaspulus flexuosus* (2 2/1 3/0 2); *Pneumolaelaps* genus, and *Melittiphis alvearius* (2 2/1 3/1 1). This means that there are some genera in laelapid mites that have two kinds of genu IV chaetotaxy with 9 or 10 setae, including one or two ventral setae. *Pogonolaelaps canestrinii*, and *Pogonolaelaps beaulieui* gen. n., sp. n. have two ventral setae on genu IV. *Gymnolaelaps* has one ventral seta on genu IV.

*Laelaspisella* can be defined as laelapid mites with the following characters:

There are two pairs of pores on the sternal shield, *iv3* apparently absent; the metasternal setae are absent; the genital shield slightly widened behind the genital setae and is rounded or somewhat tapered posteriorly, but never touching the anal shield; setae *Jv5* and *Zv5* are spatulated and pilose; the palptarsal claw two tined; chelicerae lacks dorsal seta; the anterior margin of the epistome is smooth and sharply mucronated; the whole dorsal shield with hypertrichous condition; the setation of genu I is deficient for one postero-dorsal seta (2 3/2 2/1 2); the chaetotaxy of genu IV deviates from the normal (2 2/1 3/0 1) due to the presence of a postro-ventral seta (2 2/1 3/1 1). Finally, according to the explanations above the genus *Laelaspisella* comprises two species *Laelaspisella macrodorsalis* and *Laelaspisella epigynialis* at present.

### Notes

#### Gymnolaelaps tonsilis

Taxon classificationAnimaliaMesostigmataLaelapidae

(Karg, 1989)

Pseudoparasitus (Gymnolaelaps) tonsilis Karg, 1989: 335.
Gymnolaelaps tonsilis . — Farrier and Hennessey 1993: 74; Moreira 2014: 281.

##### Specimens examined.

*Pseudoparasitus tonsilis* Karg, 1989, Chel. Nr. 3947♂, paratypus, ZMB Kat. Nr. 41478, St. Lucia, Antillen, Gastries, Vigie, Point Eins.: Dr. Mahunka, Budapest, 21 .7.80; Chel. Nr. 3944♀, ZMB Kat. Nr. 41475 (paratypes); Nr. 3945♀, ZMB Kat. Nr. 41476 (paratypes); Nr. 4440 ♀, ZMB Kat. Nr. 42589 (holotypus); Nr. 3943♀, ZMB Kat. Nr. 41474 (paratypes); Nr. 3946♂, ZMB Kat. Nr. 41477 (paratypes): with the same data as above on 11.7.80.

#### Gymnolaelaps kabitae

Taxon classificationAnimaliaMesostigmataLaelapidae

Bhattacharyya, 1968

Gymnolaelaps kabitae Bhattacharyya, 1968: 537.
Pseudoparasitus (Gymnolaelaps) kabitae . — Karg 1989: 334.
Laelaspisella kabitae . — Joharchi and Halliday 2013: 47; Moreira 2014: 317.

##### Specimens examined.

Ghaletol, Khuzestan province, nest materials of unknown ant, two females and two males, 2012-2013, coll. A. Nemati; Shahrekord, Chaharmahal va Bakhtiari province, nest materials of unknown ant, one female, 2014, coll. A. Nemati; Izeh,, Khuzestan province, nest of *Pheidole pallidula* (Hym., Formicidae), two females, one male, coll. A. Nemati. All specimens were deposited in APAS.

Psedoparasitus (Gymnolaelaps) tonsilis Karg, 1989 and *Gymnolaelaps kabitae* Bhattacharyya, 1968 possess denticulate epistome, two tined apotele, internal malae normal and lack very elongate setae and barbed, chelicera with dorsal seta, sternal shield with *iv1-3*, lack *st4*, genital shield rounded posteriorly and bear one pair of setae, ventral setae acicular, with hypertrichous condition in whole dorsal shield (based on personal observation of first author on type materials of *Gymnolaelaps tonsilis* and in spite of its original description in Karg 1989) and with simple acicular setae, males with separate sternito-genital and anal shields and without spine like setae on leg II, genu IV with two ventral setae (*av* and *pv*), genu I with three *pd* setae (*pd1-3*). Those are differed from *Laelaspisella* by having denticulate epistome, presence of dorsal seta on fixed digit of chelicera, genu I with *pd1-3*, opisthogastric setae simple acicular, internal malae without elongate setae. These species also differed from *Pogonolaelaps* gen. n. by having simple acicular dorsal setae, hypertrichy on whole dorsal shield, denticulate epistome, two tined apotele, and the absence of spine like setae on leg II of male, internal malae without very elongate setae.

## Supplementary Material

XML Treatment for Pogonolaelaps

XML Treatment for Pogonolaelaps canestrinii

XML Treatment for Pogonolaelaps beaulieui

XML Treatment for Gymnolaelaps tonsilis

XML Treatment for Gymnolaelaps kabitae
